# High-Throughput Phenotyping to Detect Drought Tolerance QTL in Wild Barley Introgression Lines

**DOI:** 10.1371/journal.pone.0097047

**Published:** 2014-05-13

**Authors:** Nora Honsdorf, Timothy John March, Bettina Berger, Mark Tester, Klaus Pillen

**Affiliations:** 1 Chair of Plant Breeding, Institute of Agricultural and Nutritional Sciences, Martin-Luther University Halle-Wittenberg, Halle (Saale), Germany; 2 Interdisciplinary Center for Crop Plant Research (IZN), Halle (Saale), Germany; 3 School of Agriculture, Food and Wine, University of Adelaide, Waite Campus, Adelaide, Australia; 4 The Plant Accelerator, University of Adelaide, Waite Campus, Adelaide, Australia; 5 Center for Desert Agriculture, King Abdullah University of Science and Technology, Thuwal, Saudi Arabia; Nanjing Agricultural University, China

## Abstract

Drought is one of the most severe stresses, endangering crop yields worldwide. In order to select drought tolerant genotypes, access to exotic germplasm and efficient phenotyping protocols are needed. In this study the high-throughput phenotyping platform “The Plant Accelerator”, Adelaide, Australia, was used to screen a set of 47 juvenile (six week old) wild barley introgression lines (S42ILs) for drought stress responses. The kinetics of growth development was evaluated under early drought stress and well watered treatments. High correlation (r = 0.98) between image based biomass estimates and actual biomass was demonstrated, and the suitability of the system to accurately and non-destructively estimate biomass was validated. Subsequently, quantitative trait loci (QTL) were located, which contributed to the genetic control of growth under drought stress. In total, 44 QTL for eleven out of 14 investigated traits were mapped, which for example controlled growth rate and water use efficiency. The correspondence of those QTL with QTL previously identified in field trials is shown. For instance, six out of eight QTL controlling plant height were also found in previous field and glasshouse studies with the same introgression lines. This indicates that phenotyping juvenile plants may assist in predicting adult plant performance. In addition, favorable wild barley alleles for growth and biomass parameters were detected, for instance, a QTL that increased biomass by approximately 36%. In particular, introgression line S42IL-121 revealed improved growth under drought stress compared to the control Scarlett. The introgression line showed a similar behavior in previous field experiments, indicating that S42IL-121 may be an attractive donor for breeding of drought tolerant barley cultivars.

## Introduction

Barley (*Hordeum vulgare* ssp. *vulgare*, hereafter abbreviated with *Hv*) is ranked fourth among the worldwide production of cereals. Due to its multipurpose use as animal feed, human food and substrate for malting it is one of the most important cereals world-wide [1]. Barley is known to be relatively tolerant to abiotic stresses among the major cereal crops and, thus, is often grown in more marginal sites [2]. However, the process of genetic erosion has been under way in barley since its domestication some 10,000 years ago and, in particular, since the advent of intensive modern elite breeding during the last century [3]. As a result of this process, diverse landraces have been replaced by modern elite cultivars with a much narrower gene pool. Therefore there is limited genetic diversity remaining in the elite barley gene pool for abiotic and biotic stress tolerance. The current loss of genetic variation in the elite gene pool tends to limit the breeding success of improved cultivars [4]. To overcome this problem several authors, e.g. Zamir [5], proposed to use wild relatives of crop species as donors of exotic germplasm to enhance elite varieties. Tanksley and Nelson [6] proposed the method of “advanced backcross quantitative trait loci analysis” (AB-QTL) to introduce exotic genes into modern crop varieties. The method combines QTL detection and the introduction of favorable exotic alleles from a wild donor parent. Lines produced by advanced backcrosses ideally contain only one single introgression from the wild parent and are then referred to as introgression lines (ILs). This is achieved by several rounds of backcrossing to the recurrent parent and marker assisted selection (MAS). A set of ILs ideally represents the whole genome of a wild donor plant in the genetic background of a single elite variety [5].

Pillen et al. [7] published the first AB-QTL study in barley. Von Korff et al. [8] developed a BC_2_DH population from a cross between the German spring barley cultivar Scarlett (*Hv*) and the Israeli wild barley accession ISR42-8 (*Hordeum vulgare* ssp. *spontaneum*, hereafter abbreviated with *Hsp*). The lines of this S42 population were used in several AB-QTL studies to identify QTL for yield, pathogen resistance and malting quality traits [9]–[13].

Schmalenbach et al. [14] used the S42 population to develop 59 ILs (S42ILs) by a further round of backcrossing with the recurrent parent Scarlett and subsequent selfing and MAS. Each of the S42ILs contains a single or a small number of *Hsp* introgressions. Several QTL studies were conducted to verify QTL from AB-QTL studies and to identify new QTL for pathogen resistance, yield and quality parameters [14]–[16]. Naz et al. [17] studied root architecture of S42ILs and detected QTL for root dry weight and root volume. Later on, the S42IL population was extended to 73 lines and the lines were genotyped with a 1,536-SNP Illumina BOPA1 set [18]. Six hundred thirty-six informative SNPs and their known map order [19] allowed the precise localization of the *Hsp* introgressions. The S42IL set represents 87.3% of the wild barley donor genome. Moreover, Schmalenbach et al. [18] developed segregating high-resolution mapping populations (S42IL-HRs) for 70 S42ILs. Those lines are readily available to facilitate fine mapping and, ultimately, cloning of QTL.

Drought is one of the main factors limiting yield worldwide [20]. Due to climate change extreme weather events are predicted to occur more frequently and an altered pattern of drought occurrence is expected [21]. Therefore maintaining plant growth and yield under drought remains a major objective for plant breeding [22]. Many studies have been conducted on the impacts of terminal drought stress in cereals, while impacts of drought stress at early developmental stages are less well investigated [23]. However, several authors comment that yield may be enhanced by improved early vigor and a rapid development of maximum leaf area [24], [25]. López-Castañeda and Richards [26] reported that on average barley has a higher yield in water limited environments compared to wheat, triticale, and oat. As part of a possible explanation, they pinpointed the faster and more vigorous growth of barley during vegetative development. Variation in this trait is, therefore, likely to be in direct relation to drought stress tolerance and yield.

Conventional methods to determine biomass and measure growth are time-consuming and labor intensive. Often they involve destructive harvest of plants and therefore make repeated measurements on the same plant impossible. New developments in plant imaging technologies allow the estimation of biomass and growth parameters as a non-destructive and rapid alternative to more traditional methods [27], [28]. New phenotyping facilities enable automated imaging of plants. Several types of plant images can be taken, e.g. with infrared, near infrared, fluorescent and visible light. Scanning with infrared light gives information on plant or leaf temperature, while near infrared imaging sheds light on the plant water content and fluorescent pictures enable conclusions on plant health status. High resolution color pictures (RGB pictures), taken from the top and two side views are used to determine the projected shoot area of the plant. The projected shoot area serves as a measure for biomass. Hence, from RGB images taken at several time points, growth curves as well as growth rates can be calculated.

In fully automated greenhouses plants can be delivered via conveyor belts to watering, weighing and imaging stations. In these high-throughput phenotyping facilities several hundred individual plants can be imaged per day in a fully automated manner. High-throughput phenotyping facilities of this type are currently in use in various research institutes (e.g. The Plant Accelerator, Adelaide, Australia; CropDesign, Gent, Belgium; IPK Gatersleben, Germany, PhenoArch, Montpellier, France).

Such phenotyping facilities are ideal to combine controlled irrigation and phenotyping protocols [29]. A first application was given by Rajendran et al. [30] who used a manual imaging system (LemnaTecScanalyzer3D, Wuerselen, Germany) to screen *Triticum monococcum* accessions for salinity tolerance. They developed high-throughput quantification assays to distinguish sodium exclusion, sodium tissue tolerance and osmotic tolerance as the strategies plants use to establish salinity tolerance.

In this report, “The Plant Accelerator” was used to screen growth of wild barley ILs under well watered and drought treatments during vegetative growth. The aims were (1) to identify wild barley derived QTL within the set of S42ILs that control drought stress responses and (2) to test the use of non-destructive high-throughput imaging to measure vegetative stage drought response in barley.

We could show that high-throughput imaging provides accurate estimates for biomass development over time. Moreover several drought related QTLs were identified and genotypes detected that may be beneficial in future breeding programs.

## Materials and Methods

### Plant Material

Forty-seven wild barley ILs of the S42IL library and the recipient parent Scarlett were selected for the experiment. The S42ILs are derived from a cross between the German malting barley variety Scarlett and the Israeli wild barley accession ISR42-8. The 47 ILs possess few *Hsp* chromosome segments and were selected based on SSR and SNP genotyping to represent a large portion, 87.3%, of the ISR42–8 genome [18]. Repeated backcrossing and MAS are described in Schmalenbach et al. [14].

### Glass House Cultivation

**Figure 1:**
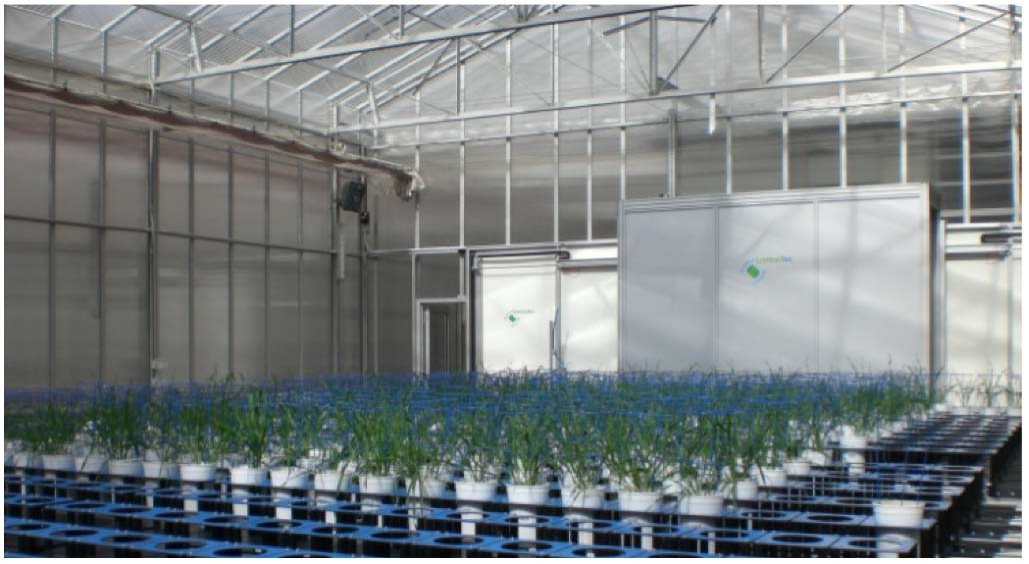
View of experiment 1 with five-week old barley S42IL plants growing in The Plant Accelerator.

Two drought stress experiments, with duration of six weeks each, were conducted between end of March and mid of July 2011 in The Plant Accelerator greenhouse facilities in Adelaide, Australia (34°58′16.18″S; 138°38′23.88″E). Forty-eight barley genotypes were grown under a well watered and stress treatments with three replicates per genotype and treatment. Each experiment was designed in three randomized blocks. Control and stress treatments of each genotype were placed next to each other (Fig. 1, Table S1).

**Figure 2:**
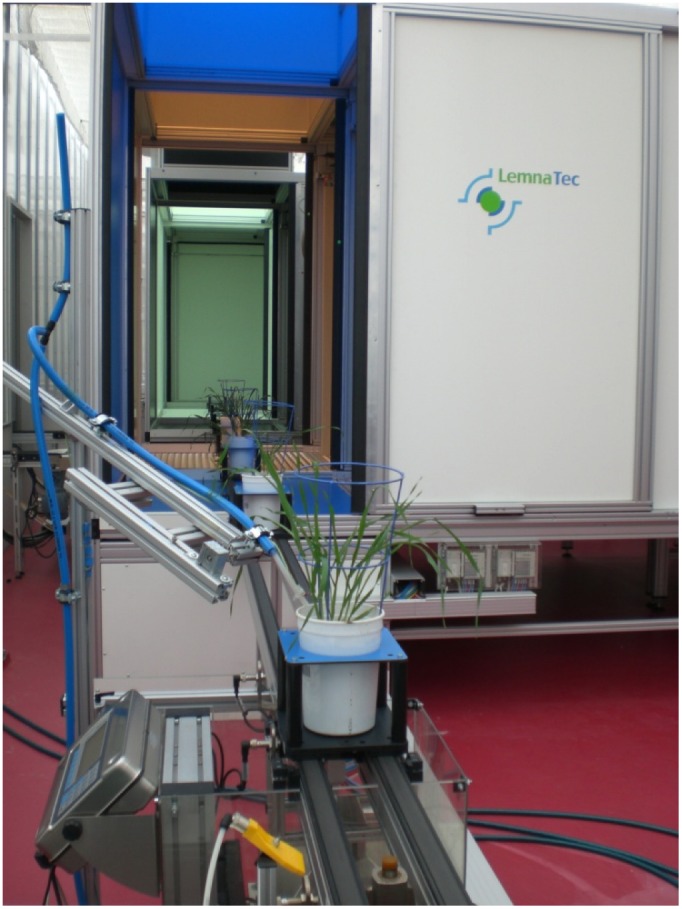
Barley plants at the weighing and watering unit after leaving the imaging station.

Single plants were grown in 2.5 L plastic containers with 2.1 kg of soil (50% UC Davis soil mix, 35% Coco-peat, 15% clay-loam). Three seeds per pot were directly sown into the soil and after germination thinned out, leaving one plant per pot. Plants were pre-grown for two weeks in a regular greenhouse and watering was performed manually to allow optimal germination and seedling establishment. Subsequently, the pots were transferred to the “smart house” where each pot was placed onto a cart on a conveyor belt and the two treatments were applied. Every second day, pots were weighed and watered automatically to 22% gravimetric water content for the well watered treatment and 15% for the stress treatment (Fig. 2). Based on the experience of the first experiment we adjusted the drought stress in the second experiment to 12% gravimetric water content to slightly increase drought effects. The experiments were carried out under natural lighting with the temperature in the greenhouse kept at a range between 15°C (night) and 22°C (day).

### Phenotyping

With the onset of the stress treatment imaging of the plants started. Plant images were captured using a LemnaTec 3D Scanalyzer (LemnaTec, GmbH, Wuerselen, Germany). Every day, three RGB pictures (2056×2454 pixels) were taken of each barley plant, one top view image and two side view images with a 90° horizontal rotation. After background-foreground separation was applied to separate the plant tissue area from the background, pixel numbers per plant were counted and the pixel sum of the three pictures per plant was taken to define the projected shoot area. The shoot area measured over time was used to draw growth curves. For each growth curve, curve fitting with a 6^th^ order polynomial was conducted to adjust for possible missing data points and absolute growth rate [dA/dt] and relative growth rate [(dA/dt)/A] were calculated. For each of the three curves the integral was determined and used as a trait in the statistical analysis. Moreover, six further traits were extracted from the images; caliper length, height, color (as hue angle in the HSI color scheme) and the two parameters shoot area top view and convex hull area to calculate compactness of each plant. At the end of the experiment, barley plants were harvested and above ground biomass, tiller number (TIL), and plant height (HEI) were determined. Fresh biomass was weighed and, subsequently, oven dried to constant weight to determine dry biomass. Water use efficiency (WUE) was calculated by dividing dry biomass at the end of the experiment by the total amount of water added during the four weeks in the “smart house” [mg/g water]. Specific plant weight (SPW) was calculated from the dry weight and the maximum projected shoot area at the end of the experiment. In addition, simple stress indices (SSI) were calculated as follows: SSI = Ts/Tc, where Ts and Tc are the average trait performances of an IL under stress and control conditions, respectively. An overview of trait definitions is given in Table 1.

**Table 1:** List of evaluated traits.

Trait	Abbreviation	Unit	Method of measurement
*Imaging parameters*			
Shoot area integral^a^	SAI	kPix^b^	Calculated from pixel sum of three images per plant per day; A
Absolute growth rate integral	AGRI	kPix/d	Calculated from pixel sum of three images per plant per day; dA/dt
Relative growth rate integral	RGRI	d^−1^	Calculated from pixel sum of three images per plant per day; (dA/dt)/A
Height integral	HEII	kPix	Max. distance from bottom to top of plant
Caliper length integral	CALI	kPix	Max. distance between two points on the object boundary, top view image
Hull area integral	HULI	kPix	Smallest geometrical object without concave parts that covers whole plant, top view image
Shoot area top view integral	SATVI	kPix	Pixel number
Plant hue integral	HUEI	-	Average hue value calculated from all pixels per plant and day
*Harvest parameters*			
Tiller number	TIL	-	Number of tillers per pot
Height	HEI	cm	Plant height measured from bottom to leaf tip
Biomass dry	BMD	g	Weight of oven dried biomass per pot
*Indices*			
Water use efficiency	WUE	mg/g water	Harvested biomass per plant/total amount of irrigation water
Specific plant weight	SPW	mg/kPix	Harvested biomass per plant/pixel number per plant at end of experiment
Compactness integral	COMI	-	SATV/HUL per plant per day
Simple stress index	SSI	-	Trait performance under stress/trait performance under control treatment

a Integral: calculated for length of entire experiment, respectively.

b kilo Pixel.

### Genotyping

The S42ILs were genotyped with the 1,536-SNP barley BOPA1 set [19] of the Illumina GoldenGate assay [18]. Six hundred and thirty-six out of the tested 1,536 SNPs were polymorphic and used to characterize the extent of exotic *Hsp* introgressions in each S42IL (see Fig. S1).

### Statistical Analysis

Statistical analyses were performed with SAS Enterprise Guide 4.2. [31]. Descriptive statistical parameters (Table S2) were calculated with procedure MEANS. Heritabilities across treatments were calculated as 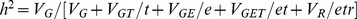 , and within treatments as: 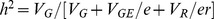. The terms V_G,_ V_GT,_ V_GE,_ V_GET_ and V_R_ represent the genotypic, genotype×treatment, genotype×environment, genotype×environment×treatment, and error variance components, respectively, calculated with procedure VARCOMP [31]. The terms t, e, and r indicate the number of treatments, experiments and replicates, respectively. Pearson correlation coefficients between traits were calculated with means across treatments, blocks and experiments and within drought stressed and control treatments, respectively, using the procedure CORR.

Analysis of variance was carried out with the procedure MIXED using model I to test for genotype main effects across treatments and experiments.

Model I:
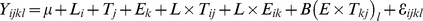



and model II for genotype effects across experiments but within a single treatment.

Model II:
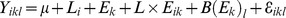
Where *µ* is the general mean, *L_i_* is the fixed effect of the *i*th line, *T_j_* is the fixed effect of the *j*th treatment, *E_k_* is the fixed effect of the *k*th experiment, *L*×*T_ij_* is the fixed interaction between the *i*th line and the *j*th treatment, *L*×*E_ik_* is the fixed interaction between the *i*th line and the *k*th experiment, *B(E*×*T_kj_)_l_* is the random effect of the *l*th block nested in the interaction between *k*th experiment and *j*th treatment, *B(E_k_)_l_* is the random effect of the *l*th block nested in the *k*th experiment and ε*_ijkl_* and ε*_ikl_* are the error of *Y_ijkl_* and *Y_ikl_,* respectively.

Following the mixed model analysis a Dunnett test was conducted where least square means (LSMEANS) of each IL were compared to the control Scarlett. In case an IL revealed a significant (P<0.05) deviation in trait performance from Scarlett, as main effect and/or as line×treatment interaction, a line×trait association was assumed and the presence of a QTL was accepted. If several ILs with overlapping introgressions showed a similar effect, it was assumed that the ILs contained the same QTL. We consider this QTL as the most likely location of the effect and, thus, define a minimum number of QTL needed to explain all identified trait effects. The relative performance (RP) of an IL was calculated as RP (IL)  =  [LSMEANS (IL) – LSMEANS (Scarlett)] ×100/LSMEANS (Scarlett), where LSMEANS were calculated with model I across treatments, experiments and blocks or with model II across experiments and blocks, separately for each treatment. The detection of significant line by trait associations was conducted for every trait revealing a heritability with h^2^>0% across treatments or within the two watering treatments, respectively.

## Results

### Trait Performance of S42ILs

**Figure 3:**
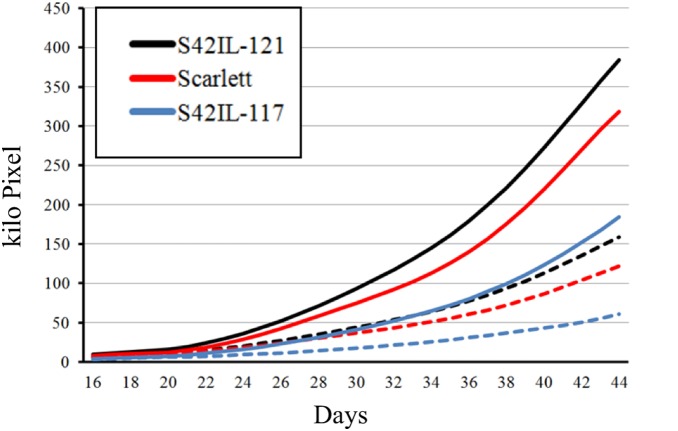
Development of shoot area of S42IL-121, Scarlett and S42IL-117 under well watered (solid line) and drought (dashed line) treatment, respectively.

**Figure 4:**
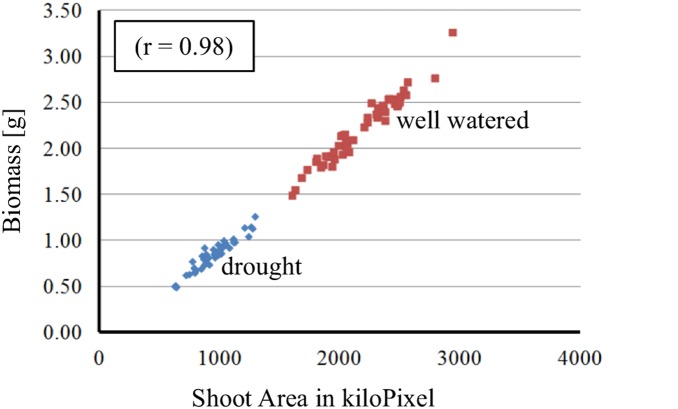
Correlation between biomass and shoot area integral under drought (blue dots) and well watered (red dots) treatment, respectively.

For most traits, means were higher under well watered treatment than under drought stress (Fig. 3 and Fig. 4, Table S2), e.g. 2.2 g of biomass dry (BMD) vs. 0.9 g. There were, however, four exceptions. Compactness integral (COMI) was higher under drought treatment than under well watered treatment. The same was true for plant hue integral (HUEI), SPW, and WUE but differences were marginal.

Coefficients of variation (CV) differed strongly between traits (1.5 to 72.1%). Highest CV was calculated for BMD across treatments (72.1%). The lowest CVs were determined for HUEI, varying from 1.5 to 1.6% for the different treatments and the SSI. CV was generally higher under drought than under well watered treatment. The four exceptions were WUE, SPW and height integral (HEII) where CV under well watered treatment was higher and HUEI where CV was the same under both treatments.

Heritability was generally higher under well watered treatment. HEI, HEII and WUE were exceptions and showed higher heritabilities under drought treatment. Highest heritabilities were found for HEI, HEII and caliper integral (CALI) (between 46.2 and 76.8%). Low heritabilities were determined for WUE and HUEI under drought treatment and across treatments, as well as for BMD under drought treatment (15%). Most of the SSI showed heritabilities equal to 0. SSI (HEII), SSI (HUEI), SSI (SPW), SSI (WUE) revealed heritabilities between 4.7 and 14.5%.

### Trait Correlations

Highest correlations were found among measured traits and among stress indices (Table S3). However, correlations between measured traits and stress indices were low. The measured traits showed the highest correlations between shoot area integral (SAI) and absolute growth rate integral (AGRI) (r = 0.99), BMD and SAI (Fig. 4), BMD and AGRI, shoot area top view integral (SATVI) and SAI and SATVI and AGRI (r = 0.98). Most correlations were positive and statistically significant. HEI showed negative correlation with relative growth rate integral (RGRI) and TIL, but values were not statistically significant. COMI showed negative correlations with all traits but HEII. Among stress indices highest correlations were found for SSI (SAI), SSI (AGRI) and SSI (SAI), as well as between SSI (WUE) and SSI (SPW) (with r>0.93). Interestingly, WUE, SPW and BMD showed only low correlations (r<0.43). Looking at the simple stress index, however, correlations between SSI (WUE), SSI (SPW) and SSI (BMD) were very high (r = 0.85 to 0.96). Autocorrelations between drought and well watered treatments of a single trait were high (r>0.61) for most traits. HEI with r = 0.85 had the highest correlation between treatments. RGRI showed a low but still significant correlation between the treatments with r = 0.33. Autocorrelations for SPW and WUE were not significant.

### Mixed Model Analysis of Variance

The mixed model analysis, including fixed line, treatment, and experiment effects (i.e. model I) revealed significant (P<0.05) line effects for all investigated traits (Table S4). Treatment had a clear impact on trait performance. For all traits, except WUE and SPW the effect was significant. Line by treatment interaction effects were not significant for any of the measured traits. The experiments had a significant effect on trait performance of all traits except leaf color measured as plant hue integral (HUEI). And line by experiment interaction was significant for all traits but RGRI, COMI, and HEII. In the mixed model analyses for single treatments including fixed line, and experiment effects (i.e. model II), line had a significant effect on trait performance for all traits but HUEI, RGRI, and WUE under well watered and HUEI and SPW under drought conditions (Tables S5 and S6). Also simple stress indices were analyzed with model II (Table S7). The line effect was not significant for any of the simple stress indices.

### QTL Detection

**Figure 5:**
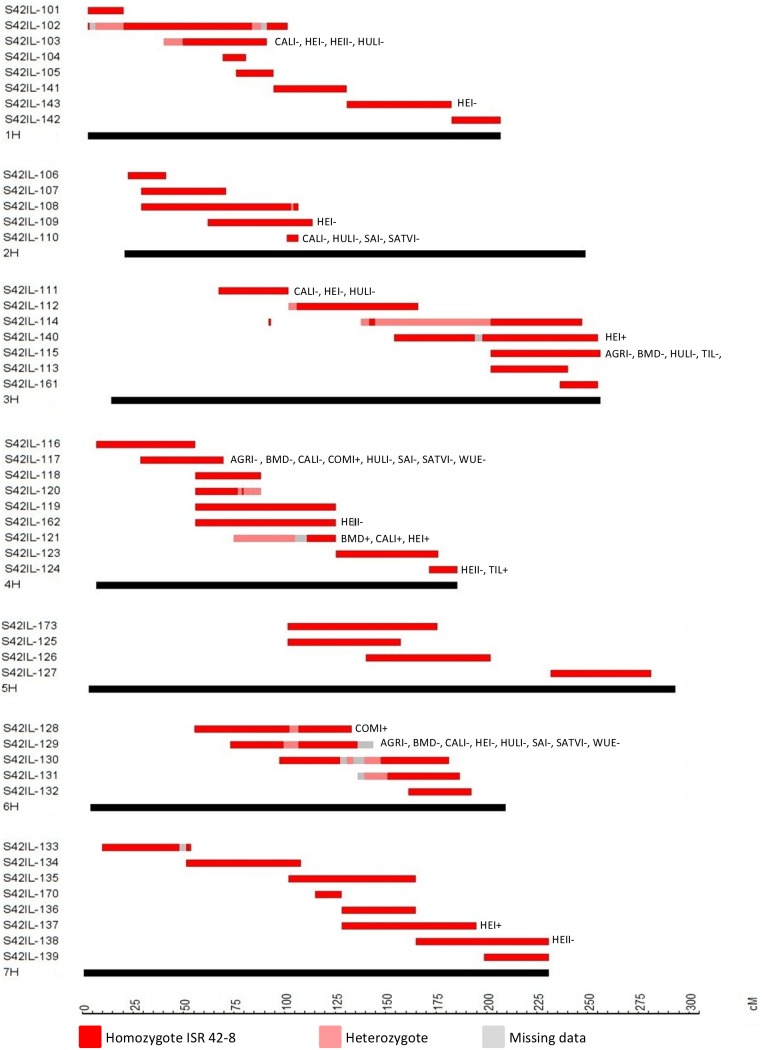
QTL map with indication of S42IL introgressions (Schmalenbach et al. 2011). SNP positions (in cM) are based on Close et al. (2009). QTL are placed right to the S42IL, indicated by trait abbreviations (see Table 1). The sign indicates an increasing (+) or a decreasing (−) *Hsp* effect.

QTL were only determined for traits with heritability greater than 0. The Dunnett tests revealed, in total, 63 line effects for eleven out of 14 traits. These effects were detected either across treatments (39), within the drought treatment (15) or within the well watered treatment (9). Several of the measured effects were consistent between the different treatments. Thus, these line effects were summarized to a minimum of 44 QTL (Table 2 and Fig. 5). No QTL were identified for RGRI, SPW, and HUEI. Between two and nine QTL were identified for the traits AGRI, BMD, CALI, COMI, HEI, HEII, hull area integral (HULI), SAI, SATVI, TIL, and WUE. In the following, the QTL are presented for each trait separately.

**Table 2:** List of 44QTL detected for 11 traits in the S42IL-population.

Trait^a^	QTL Name	Position of main introgression^b^	Line	Treatment^c^	LSMEANS Scarlett^d^	LSMEANS IL^e^	Dev. f. Sca	Dev. f. Sca %^g^	Candidategenes^h^	Studies withcorresponding QTL^i^
AGRI	QAgri.S42IL-3H	3H; 204.48–255.13	S42IL-115	a	170.84	119.10	−51.74	−30.28		
	QAgri.S42IL-4H	4H; 027.52–064.77	S42IL-117	a, d	170.84	114.69	−56.15	−32.87		
	QAgri.S42IL-6H	6H; 073.90–133.47	S42IL-129	a	170.84	120.89	−49.95	−29.24		
BMD	QBmd.S42IL-3H	3H; 204.48–255.13	S42IL-115	a	1.66	1.10	−0.56	−33.64		
	QBmd.S42IL-4H	4H; 027.52–064.77	S42IL-117	a	1.66	0.99	−0.67	−40.30		
	QBmd.S42IL-4Hb	4H; 074.11–119.06	S42IL-121	a	1.66	2.26	0.60	35.97		
	QBmd.S42IL-6H	6H; 073.90–133.47	S42IL-129	a	1.66	1.10	−0.57	−33.94		IV
CALI	QCali.S42IL-1H	1H; 040.51–089.01	S42IL-103	a, w	19.39	15.83	−3.56	−18.35		
	QCali.S42IL-2H	2H; 102.66–104.81	S42IL-110	a, d	19.39	15.47	−3.92	−20.23		
	QCali.S42IL-3H	3H; 067.01–098.41	S42IL-111	a	19.39	16.52	−2.87	−14.80		
	QCali.S42IL-4H	4H; 027.52–064.77	S42IL-117	a, d	19.39	15.98	−3.42	−17.62		
	QCali.S42IL-4Hb	4H; 074.11–119.06	S42IL-121	a	19.39	22.34	2.94	15.18		
	QCali.S42IL-6H	6H; 073.90–133.47	S42IL-129	a, d, w	19.39	15.02	−4.37	−22.55		
COMI	QComi.S42IL-4H	4H; 027.52–064.77	S42IL-117	a, w	4.10	5.32	1.22	29.77		
	QComi.S42IL-6H	6H; 071.39–132.23	S42IL-128	w	3.82	5.23	1.42	37.13		
HEI	QHei.S42IL-1H	1H; 040.51–089.01	S42IL-103	a, w	49.00	42.75	−6.25	−12.76		
	QHei.S42IL-1Hb	1H; 130.68–173.49	S42IL-143	a	49.00	43.50	−5.50	−11.22	*HvFT-3^3^*	I
	QHei.S42IL-2H	2H; 063.96–110.84	S42IL-109	a	49.00	44.33	−4.67	−9.52	*sdw3^2^, HvFT4^3^*	I, II, III, IV
	QHei.S42IL-3H	3H; 067.01–098.41	S42IL-111	a	49.00	44.50	−4.50	−9.18		
	QHei.S42IL-3Hb	3H; 154.99–253.73	S42IL-140	a, d, w	49.00	58.17	9.17	18.71	*denso^1^*	I, IV, V
	QHei.S42IL-4H	4H; 074.11–119.06	S42IL-121	a, d, w	49.00	57.75	8.75	17.86		II, IV
	QHei.S42IL-6H	6H; 073.90–133.47	S42IL-129	a	49.00	44.42	−4.58	−9.35		V
	QHei.S42IL-7H	7H; 134.43–193.89	S42IL-137	a, d	49.00	54.67	5.67	11.56		I, II, IV, V
HEII	QHeii.S42IL-1H	1H; 040.51–089.01	S42IL-103	a	11.67	9.85	−1.83	−15.64		
	QHeii.S42IL-1Hb	1H; 130.68–173.49	S42IL-143	a	11.67	10.06	−1.62	−13.85	*HvFT-3^3^*	I
	QHeii.S42IL-4H	4H; 061.15–119.06	S42IL-162	a	11.67	9.99	−1.68	−14.39		
	QHeii.S42IL-4Hb	4H; 171.25–183.54	S42IL-124	a	11.67	10.04	−1.64	−14.02		
	QHeii.S42IL-7H	7H; 176.37–229.66	S42IL-138	a	11.67	10.05	−1.63	−13.93		
HULI	QHuli.S42IL-1H	1H; 040.51–089.01	S42IL-103	a, w	6679	4443	−2236	−33.47		
	QHuli.S42IL-2H	2H; 102.66–104.81	S42IL-110	a, d	6679	4517	−2161	−32.36		
	QHuli.S42IL-3H	3H; 067.01–098.41	S42IL-111	a	6679	4617	−2062	−30.88		
	QHuli.S42IL-3Hb	3H; 204.48–255.13	S42IL-115	a	6679	4616	−2063	−30.89		
	QHuli.S42IL-4H	4H; 027.52–064.77	S42IL-117	a	6679	4327	−2352	−35.21		
	QHuli.S42IL-6H	6H; 073.90–133.47	S42IL-129	a	6679	4179	−2500	−37.42		
SAI	QSai.S42IL-2H	2H; 102.66–104.81	S42IL-110	d	995	625	−369	−37.14		I, (as biomass)
	QSai.S42IL-4H	4H; 027.52–064.77	S42IL-117	a, d	1654	1116	−538	−32.53		
	QSai.S42IL-6H	6H; 073.90–133.47	S42IL-129	a, d	1654	1151	−504	−30.44		
SATVI	QSatvi.S42IL-2H	2H; 102.66–104.81	S42IL-110	d	398	214	−184	−46.18		
	QSatvi.S42IL-4H	4H; 027.52–064.77	S42IL-117	a	677	437	−240	−35.44		
	QSatvi.S42IL-6H	6H; 073.90–133.47	S42IL-129	a	677	450	−228	−33.64		
TIL	QTil.S42IL-3H	3H; 204.48–255.13	S42IL-115	a	8.17	5.92	−2.25	−27.55		
	QTil.S42IL-4H	4H; 171.25–183.54	S42IL-124	a, d, w	8.17	11.67	3.50	42.86	*VRN-H2^3^*	III, V
WUE	QWue.S42IL-4H	4H; 027.52–064.77	S42IL-117	d	1.97	1.25	−0.72	−36.53		
	QWue.S42IL-6H	6H; 073.90–133.47	S42IL-129	d	1.97	1.17	−0.80	−40.59		

a Trait abbreviations are given in Table 1.

b By chromosome and cM position.

c Treatment under which effect occured, a: main effect, d: under drought treatment, w: under well watered treatment.

d LSMEANS Scarlett for the indicated trait and treatment.

e LSMEANS for indicated IL for indicated trait and treatment, if under more than one treatment then a.

f Deviation from Scarlett  =  LSMEANS[IL] - LSMEANS [Scarlett].

g Deviation from Scarlett in %  =  (LSMEANS[IL] – LSMEANS [Scarlett])/LSMEANS[Scarlett] *100.

h References: ^1^ Laurie et al. (1995), ^2^ Gottwald et al. (2004), ^3^ Wang et al. (2010)

i I: Von Korff et al. (2006), II: Schmalenbach et al. (2009), III: Wang et al. (2010), IV: March et al. (in prep.), V: Honsdorf et al. (in prep.).

### Absolute Growth Rate Integral (AGRI)

Three QTL were identified for AGRI. The QTL are located on chromosomes 3H, 4H and 6H and the *Hsp* allele in all three QTL reduced the trait performance. Across treatments the *Hsp* alleles reduced the integral of the absolute growth rate by approximately 30%. Under drought conditions the *Hsp* allele of QTL QAgri.S42IL-4H reduced the trait performance by almost 40%.

### Biomass Dry (BMD)

For biomass four QTL were identified across treatments. The *Hsp* alleles at QTL QBmd.S42IL-3H and QBmd.S42IL-6H on chromosomes 3H and 6H reduced biomass by approximately 33%. The two QTL QBmd.S42IL-4H and QBmd.S42IL-4Hb on chromosome 4H showed contrary effects. While at the first QTL the *Hsp* allele reduced biomass by 40.3%, at the second QTL it increased it by 36.0%.

### Caliper Length Integral (CALI)

Six QTL were detected for caliper length on chromosome 1H, 2H, 3H, 4H and 6H. All QTL were detected across treatments, three and two of them also showed effects for drought and well watered treatments, respectively. In five cases the *Hsp* allele had decreasing effects between 14.8 and 22.6% across treatments. In one case the *Hsp* allele at QTL QCali.S42IL-4Hb increased caliper length by 15.2% compared to Scarlett.

### Compactness Integral (COMI)

For COMI two QTL were detected on chromosomes 4H and 6H. The effect of the *Hsp* allele at QComi.S42IL-4H was observed across treatments and under well watered treatment. It increased compactness across treatments by 29.8%. In case of QComi.S42IL 6H the effect was solely observed under well watered treatment and lead to an increase of 27.1% by the *Hsp* allele.

### Height (HEI)

The highest number of QTL was detected for plant height. Eight QTL were detected across treatments. Of those, four also showed effects under either one or both of well watered and drought treatment. The QTL are located on all chromosomes except 5H. In five cases the *Hsp* alleles reduced plant height (9.2 to 12.8%). In three cases the *Hsp* allele increased plant height by 11.6 to 18.7%.

### Height Integral (HEII)

Five QTL across treatments were detected for HEII on 1H, 4H and 7H. Two of those, QHeii.S42IL-1H and QHeii.S42IL-1Hb, coincided with QTL for manual measurement of HEI. At all detected QTL the *Hsp* alleles reduced HEII by between 13.9 to 15.6%.

### Hull Area Integral (HULI)

For HULI six QTL were detected on chromosomes 1H, 2H, 3H, 4H, and 6H. In all cases the *Hsp* alleles reduced the hull area by between 30.9 to 37.4% across treatments. In addition, QHuli.S42IL-1H and QHuli.S42IL-2H showed effects under well watered and drought treatments, respectively.

### Shoot Area Integral (SAI)

Three QTL were found for the integral of the projected shoot area. The QTL are located on chromosomes 2H, 4H, and 6H. All three QTL were detected under drought treatment, while, in addition, QSai.S42IL-4H and QSai.S42IL-6H showed effects across treatments. The presence of the *Hsp* allele reduced the projected shoot area between 30.4 and 37.1% across treatments.

### Shoot Area Top View Integral (SATVI)

Three QTL were detected for shoot area top view that corresponded to the same QTL detected for SAI on chromosomes 2H, 4H, and 6H. However, only QSatvi.S42IL-2H was detected under drought treatment, while the two other QTL were detected across treatments. In all cases the *Hsp* alleles reduced SATVI between 33.6 and 46.2% compared to Scarlett.

### Tiller Number (TIL)

For tiller number two QTL were identified on chromosomes 3H and 4H. The *Hsp* allele at QTil.S42IL-3H reduced tiller number by 27.6% across treatments. QTil.S42IL-4H was detected across treatments and separately within the two treatments. Across both treatments the *Hsp* allele increased the tiller number by 42.9%.

### Water Use Efficiency (WUE)

Two QTL were detected for WUE. The *Hsp* allele of QTL QWue.S42IL-4H and QWue.S42IL-6H reduced WUE by 36.5 and 40.6% under drought treatment, respectively.

### Relative Growth Rate Integral (RGRI) and Plant Hue Integral (HUEI), Specific Plant Weight (SPW), and Simple Stress Index (SSI)

For RGRI, HUEI, and SPW and the SSIs no QTL were detected in this study.

## Discussion

The aim of the study was to validate the use of non-destructive high-throughput phenotyping to measure vegetative drought response in barley and to identify QTL derived from wild barley that control physiological traits related to drought stress. To the authorś knowledge this is the first QTL report on drought stress that used a high-throughput phenotyping facility.

Plant growth and the biomass parameters tiller number, plant height, and shoot dry weight of 48 barley genotypes were investigated under drought and well watered treatments. Two week old barley plants were transferred into a high-throughput phenotyping greenhouse, where stress and control treatments were applied automatically. During the following four weeks of cultivation, plants were imaged daily in an automated manner. Images were processed and used as a measure for plant height, caliper length, biomass and, consequently, plant growth, and plant color. After a total of six weeks green plants were harvested. Tiller number, plant height, and shoot dry weight were determined for each plant. Moreover, plant compactness, water use efficiency and specific plant weight, as well as stress indices were calculated.

The mixed model ANOVA revealed a clear effect of the treatment on trait expression. Drought stressed plants had a lower growth rate and subsequently produced less biomass (see Fig. 4). However, there was no significant line×treatment interaction, indicating that the S42ILs reacted similar under drought stress and well watered conditions for the investigated traits. This finding is also supported by high autocorrelations for investigation of traits under drought and control treatments, e.g. 0.77 for biomass (see Table S3).

### QTL Detection

In this study 44 QTL were identified in 15 ILs for eleven traits. In eight cases the *Hsp* alleles increased the performance of the trait, while in 36 cases there was a decreasing effect. This is to be expected since wild barley is known to carry many unfavorable alleles as well [5]. In six ILs only one QTL was determined, predominantly for HEI and HEII. Multiple QTL effects were found in nine ILs. For S42IL-115,-117, -121, and -129 QTL for BMD were detected in combination with one or more of the traits AGRI, HEI, SAI, and TIL which is in agreement with the high correlations found between those traits. SAI is a measure for biomass, and AGRI is the first derivative of SAI. Therefore, it was expected that ILs show effects for all three traits, simultaneously. However, this was not always the case. Thus, increasing the number of experiments and replications might be useful to increase the power of QTL detection. In the following, the traits or trait complexes are discussed separately.

### Absolute Growth Rate Integral, Shoot Area Integral and Biomass Dry (AGRI, SAI, BMD)

The three traits were highly correlated with each other (r = 0.98 and r = 0.99). Since their relation may be functional, it appears likely that a single pleiotropic QTL may control the three traits AGRI, SAI and BMD, simultaneously.

For line S42IL-129 a biomass reduction of 33.9% was observed. March et al. (in prep.) found a similar decrease in biomass in that line measured under terminal drought stress. This suggests that biomass production may be partly controlled by similar genes during early and late drought stress occurrence.

Three QTL were detected for SAI. Two of those, namely QSai.S42IL-4H and QSai.S42IL-6H, in line S42IL-117 und S42IL-129, respectively, were in accordance with BMD QTL. The *Hsp* allele, in both lines caused a decrease in the projected shoot area. Due to the high correlation of the traits it can be assumed that QTL for biomass correspond to QTL for shoot area. The *Hsp* allele of the QTL QSai.S42IL-2H on chromosome 2H caused a decrease of 37% in projected shoot area, compared to Scarlett. Von Korff et al. [10] described a QTL related to biomass reduction (QMas.S42-2H.a) in the same region in an AB-QTL field trial. Since this AB-QTL population is the parent population of the S42ILs used in this study, it is likely that the same QTL was detected in both the greenhouse and field trials.

AGRI is directly related to SAI. This might be seen as a reason for the detection of similar QTL for AGRI and SAI and, consequently, BMD. Many QTL studies on growth focus on relative growth rate instead of absolute growth rate. In the present study RGRI showed only a weak correlation to AGRI (r = 0.35) and other biomass parameters. Poorter et al. [33] pointed out that in their study QTL for RGR rather co-located with QTL for seed mass than with QTL for biomass. This fits well to the weak correlations found between biomass parameters and RGRI. However, two of the QTL detected for AGRI coincided with locations where previous studies mapped QTL for RGR. Yin et al. [34] reported a minor effect for relative growth rate associated with the *denso* locus on chromosome 3H in a spring barley recombinant inbred line (RIL) population of the cross Prisma×Apex, which may co-localize with QAgri.S42IL-3H. Poorter et al. [33] conducted QTL studies in a F_2_ population derived from a cross between two *Hsp* accessions. They mapped QTL for relative growth rate on chromosomes 1H, 2H, 5H and a minor QTL on 6H. The latter one might be in accordance with QAgri.S42IL-6H.

### Tiller Number (TIL)

Two QTL were detected for the trait tiller number on chromosomes 3H and 4H. Wang et al. [35] identified the *VRN-H2* gene on chromosome 4H in introgression line S42IL-124. Whereas S42IL-124 carries a dominant winter-type allele, Scarlett carries the recessive and deleted spring type allele at *Vrn-H2*. S42IL-124 showed an increased tiller number compared to Scarlett. Since *Vrn-H2* is known to have a pleiotropic effect on tiller number [36], we assume that this gene explains the underlying effect of the QTL. Studies on other populations revealed QTL for tiller number on chromosomes 4H as well. In a cross between two wild barley accessions Elberse et al. [37] found a QTL for tiller number on that chromosome. Baum et al. [38] identified a QTL on chromosome 4H where the *Hsp* allele increased the number of tillers and a QTL on chromosome 3H where the *Hsp* allele had a decreasing impact in an Arta×*Hsp* 41-1 RIL population. Those effects might correspond to the QTL detected in this study. Both QTL occurred irrespective of the treatment. Especially QTil.S42IL-4H appears to be a very stable QTL. It was detected across and within treatments and was detected in several studies under varying conditions, in field studies as well as under greenhouse conditions. Moreover, von Korff et al. [10] detected QTL for number of ears, which is directly related to tiller number, in the same genomic regions. On 4H the *Hsp* allele increased the number of ears, while on 3H it has a decreasing effect. This supports the observation of a stable QTL.

### Height (HEI) and Height Integral (HEII)

Plant height was determined in two ways. First, height (HEII) was modeled from the images taken during four weeks and the integral of the height growth curve was calculated. Second, height (HEI) was measured manually when plants were harvested after six weeks at the end of the experiment. The correlation between HEI and HEII was relatively low with r = 0.72, compared to the correlation between SAI and BMD with r = 0.98. While SAI shows a constant increase over time, HEII shows an overall increase, but fluctuation between days may be strong. When a new leaf is unfolded the plant grows higher, however, when the leaf becomes too heavy and bends down, the height of the plant appears to be shorter. At the end of the experiment the length of the stretched plant was measured, which is longer than the upright standing plant. Nevertheless two coinciding QTL were found between HEI and HEII on chromosome 1H.

Six out of eight QTL were already identified in previous field studies with the S42 population and/or the S42ILs, exhibiting similar effects of the same direction in all three studies. QTL QHei.S42IL-3Hb was already detected in von Korff et al. [10] and March et al. (in prep.). In this region also the *denso* dwarfing gene was mapped [39], which may be identical with the semi-dwarf gene *sdw1* [40]. The second largest effect, after QHei.S42IL 3Hb, was associated with QHei.S42IL-4H in S42IL-121. This QTL corresponds to QHei.S42IL-4H.a in Schmalenbach et al. [15]. In both studies the *Hsp* allele increased plant height by 18%. March et al. (in prep.) mapped a QTL for height for S42IL-121 as well. A third QTL (QHei.S42IL-7H) with an increasing effect of the *Hsp* allele was detected on chromosome 7H. Here an effect that was already found in the studies of von Korff et al. [10], Schmalenbach et al. [15] and March et al. (in prep.) could be verified. Moreover two QTL where the *Hsp* allele had a decreasing effect on plant height [10] were verified. In S42IL-143 HEI was reduced by 11% (QHei.S42IL-1Hb) and HEII (QHeii.S42IL-1Hb) by 14%. Von Korff et al. [10] detected a QTL in the same region on chromosome 1H. The flowering time gene *HvFT3* is mapped in the same region and known to have a pleiotropic effect on plant height [35]. QHei.S42IL-2H in S42IL-109 had reduced height by 9.5%. March et al. (in prep.) and Schmalenbach et al. [15] found the same effect in their studies. Von Korff et al. [10] found a similar effect in the region where the *Hsp* introgression of S42IL-109 was mapped. Moreover two candidate genes are mapped to the chromosomal region. These are the dwarfing gene *sdw3* [41] and the flowering gene *HvFT4*, which is known to have an effect on plant height [35].

All of the HEI QTL in the present experiments were detected across treatments. Six out of eight QTL were also found in previous field and glasshouse studies. The QTL therefore seem to be very stable across locations as well as across treatments. Moreover they seem to be independent of the developmental stage. The present experiments, thus, allowed the verification of effects after six weeks that were previously screened in field experiments after flowering, indicating that phenotyping juvenile plants may be predictive for adult plant performance, at least in regard to growth parameters. The high heritability of 76.8% supports this finding.

For HEII two QTL coincided with previous studies. Besides QHeii.S42IL-1Hb mentioned above, this was QHeii.S42IL-4Hb where the *Hsp* allele reduced height by 14%. This QTL was also detected by von Korff et al. [10] and Wang et al. [35]. Heritability for digitally determined height was lower (61.4%) than for the manually measured one. Determining height by multiple measurements apparently was not an advantage here. However, this may change at a later stage of plant development. After shooting, the plant height is less subjected to bending of leaves and therefore can be measured more precisely by the imaging technique.

### Water Use Efficiency (WUE)

Water use efficiency indicates how much biomass a plant can produce per unit water supplied. Thus, increased WUE has the potential to improve yield under drought stress conditions. Measuring WUE in regular greenhouse experiments is time-consuming. Therefore the high-throughput phenomics facility greatly assisted in scoring of water use efficiency through automated watering of pots to specific weights.

In this study the two S42ILs -117 and -129, with wild barley introgressions on chromosomes 4H and 6H, respectively, showed significant differences in WUE compared to Scarlett. Both ILs showed reduced water use efficiency compared to Scarlett. These ILs also produced less biomass. S42IL-117 and S42IL-129, thus, clearly carry unfavorable alleles for this trait. Chen et al. [42] pointed out that WUE itself is difficult to measure under field conditions and that a suitable tool to measure WUE efficiency is missing. Carbon isotope discrimination is a commonly used technique to measure WUE. Teulat et al. [43] used this method and identified QTL for WUE on chromosome 6H in a set of 167 RILs from a cross between Tadmor and Er/Apm and likewise Diab et al. [44] identified a QTL for the same trait on chromosome 4H. The QTL detected in this study may correspond to the ones found in the studies mentioned before and suggest the results from both techniques are correlated.

### Compactness Integral, Shoot Area Top View Integral, Hull Area Integral (COMI, SATVI and HULI)

The compactness of a plant describes how much of the hull area is covered by leaves. It was calculated as the ratio of SATVI to HULI. The more compact a plant is, the more ground cover it has with regard to the hull area. Two QTL were detected for this trait.

SATVI and HULI showed a high correlation of r = 0.9, however, correlations between COMI and HULI and between COMI and SATVI were only moderate and negative. This indicates that in general, bigger plants take more space and have a lower compactness compared to smaller plants. In the present experiments this was observed by comparing drought stressed and well watered plants. Drought stressed plants showed on average a higher compactness than well watered plants. Jansen et al. [45] report the same effect on a study in *Arabidopsis thaliana*. Compactness shows negative correlation with all other traits evaluated in this experiment, with the exception of HEII (r = 0.16). An example for this is S42IL-117. This introgression line has a higher compactness, but reduced biomass, and other growth parameters compared to Scarlett.

SATVI is one of the three parameters that control SAI and, thus, is highly correlated with this trait as well as with BMD and AGRI. As one may expect, for SATVI the same QTL were detected as for SAI. For HULI a total of six QTL were detected. Three of those may be due to high correlations in accordance with SATVI, AGRI and BMD.

### Caliper Length Integral (CALI)

Caliper length describes the maximum diameter of the plant. For this trait six QTL were detected. Those were in accordance with QTL for HULI. This can be explained by the close connection of both traits. Hull area is taken as the basis to calculate caliper length and both traits are highly correlated (r = 0.94). CALI also shows positive and high correlations with AGRI, SAI, SATVI, HEI, and BMD. Plants with a large diameter cover a larger area, tend to be bigger, have a higher growth rate and a higher biomass than plants with a smaller diameter. Therefore, a lot of information on plant structure can be deduced from the plant diameter.

### Stress Indices

Simple stress indices were calculated for each trait as the ratio of the mean plant performance under drought stress versus well watered treatments. In this study no QTL for a SSI was detected. Additionally the authors used two more complex stress indices (modified after Fischer and Maurer [46]), but were not able to detect QTL with those either. A stress index states how well a genotype performs under stress conditions relative to its performance under control conditions. Therefore, to see differences between genotypes for a stress index, a line by treatment interaction is necessary. If all genotypes show a similar growth reaction under stress and control conditions, the initially existing differences between the genotypes may be drastically reduced. In the present experiments line by treatment interactions were not significant and autocorrelations were high between the treatments. This may be the reason why no significant effect for the stress indices was found. This notion is supported by Wang et al. [47]. In their study on “mathematically-derived traits in QTL mapping” the authors pointed out that an increased complexity of the genetic architecture of derived traits (e.g. stress indices) may reduce the power of QTL detection.

### High-throughput Phenotyping using The Plant Accelerator

Determination of biomass by manual harvest is tedious and time-consuming. In addition, destructive harvest makes repeated measurements on the same plant impossible. Visual light imaging technologies applied in this study can solve these problems by utilizing the strong correlation between the projected shoot area and the actual biomass [27]. Imaging technologies have been successfully used in several studies in *Arabidopsis thaliana*, e.g. Granier et al. [48] and Leister et al. [49]. The first study investigated nine accessions under different levels of water deficit in the phenotyping facility “PHENOPSIS”. Reaction to water deficits was, amongst other traits, characterized by leaf area growth determined through images. The authors pointed out the importance of the automated watering in their experiment, which enables equal conditions for all plants. A characteristic that was also found very important in the present experiments. Leister et al. [49] described a first approach of using an image based technology for high-throughput growth analysis. They calculated plant area from top view images and found high correlations to plant fresh weight.

In this study the sum of three two-dimensional pictures was used as a measure of plant biomass. In these experiments correlation between SAI and BMD and between AGRI and BMD were very high (r = 0.98). The results with six-week old barley plants proved that the sum of three pictures accounts sufficiently for overlapping leaves during early development. Rajendran et al. [30] found the same for *T. monococcum*. However, as Munns et al. [27] pointed out, accuracy may decline when plants become larger and produce multiple shoots. The results of the present study approved that The Plant Accelerator is suited to enable detailed growth analysis of barley plants. The prediction of biomass by the image-based leaf sum gave accurate results when comparing to actual biomass. Growth curves can give detailed information on differences in development of genotypes. For instance, the maximum of the absolute growth rate gives insight into the change from vegetative to generative phase of plant development. The present experiments ran only for six weeks. Therefore not all plants have reached this point. In future experiments this factor should be accounted for by adjusting the duration of the experiment. Automated imaging and the appropriate analysis pipeline make the detection of different developmental stages of plants feasible in high-throughput. With this technique it is possible to detect differences in stress responses between genotypes not only at different time points, but also to account for differences in development at those time points.

Rajendran et al. [30] used non-destructive imaging to screen for different response mechanisms of *T. monococcum* to salt stress. In contrast to conventional salt stress experiments, where tolerance is measured as total biomass production of stressed plants compared to unstressed plants, the growth curves provided through daily imaging gave detailed insight into the tolerance mechanisms of the plants. While osmotically tolerant plants showed a constant growth rate, the growth rate of sodium excluders first dropped than increased after a couple of days. Moreover plant color was analyzed. No stress symptoms occurred on leaves in the present experiments. Due to little variation between the genotypes no QTL was detected for leave color. However, in the experiments by Rajendran et al. [30], color analysis was successfully used to screen leaf damages due to high salt concentrations.

Fluorescence imaging gives information on the health statues of a plant. It allows for detection of leaf senescence and necrosis. However, such symptoms were not observed in the present experiments and, thus, this parameter was not applied. Nonetheless, the technique is readily available. In addition, near infrared (NIR) and infrared (IR) imaging may be useful for future plant growth evaluations and QTL studies. NIR enables the observation of the water status of a plant, while IR is used to determine shoot temperature.

## Conclusion

In this study the use of a non-destructive high-throughput phenotyping platform was implemented to map QTL controlling vegetative drought stress responses in barley. Several QTL where the exotic *Hsp* allele had a positive effect on trait performance were detected. In particular, introgression line S42IL-121 showed improved growth under drought stress compared to the recurrent parent Scarlett. The line showed the same behavior in previous field experiments. Thus, this introgression line might be interesting for further breeding.

Moreover, several QTL were detected where the *Hsp* allele had a decreasing effect on trait performance. Especially two QTL for water use efficiency might be interesting for further investigation. In future, interesting effects of S42IL-121 and other S42ILs will be fine mapped with already available high-resolution progeny [18] to further narrow down the QTL region and, ultimately, clone the underlying genes, which caused the observed QTL effects.

## Supporting Information

Figure S1 **Map of 47 S42ILs, the map contains 636 BOPA1 SNPs.**
(PPTX)

Table S1 **Experimental layout with genotypes referred to positions (1–100) per replication (1–3).**
(XLSX)

Table S2 **Descriptive statistics for S42Ils.**
(XLSX)

Table S3 **Correlations between traits and stress indices.**
(XLSX)

Table S4 **ANOVA (Model I) results of studied traits.**
(XLSX)

Table S5 **ANOVA (Model II drought) results of studied traits.**
(XLSX)

Table S6 **ANOVA (Model II well watered) results of studied traits.**
(XLSX)

Table S7 **ANOVA (Model II SSI) results of studied traits.**
(XLSX)
