# Supplementary material for: High-Throughput Phenotyping to Detect Drought Tolerance QTL in Wild Barley Introgression Lines
Source: PLoS One. 2014 May 13;9(5):e97047. doi: 10.1371/journal.pone.0097047 (PMC4019662; doi:10.1371/journal.pone.0097047)
Supplement: Figure S1 — Map of 47 S42ILs, the map contains 636 BOPA1 SNPs. (PPTX) [file pone.0097047.s001.pptx]

## Slide 1
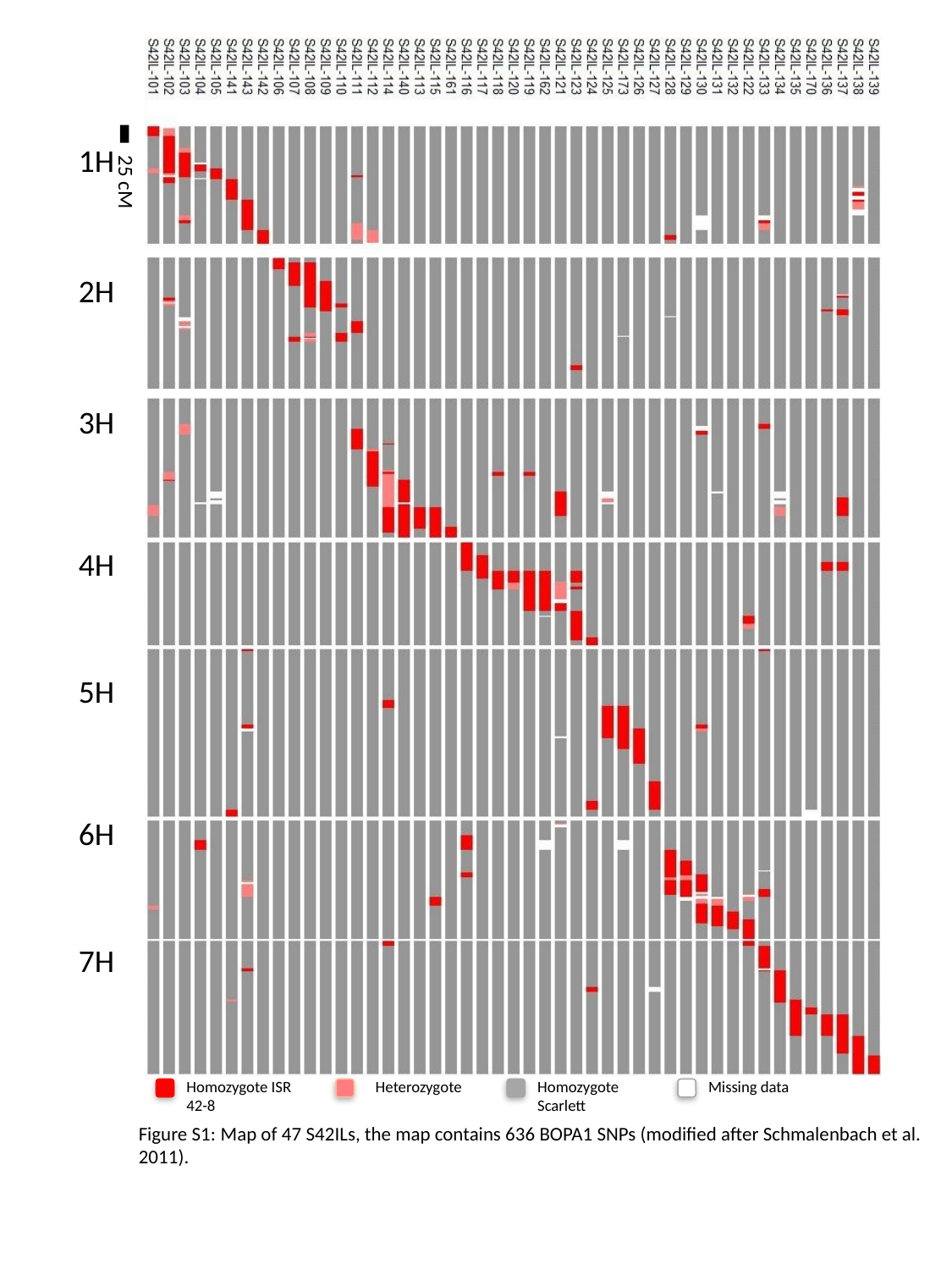

1H
25 cM
2H
3H
4H
5H
6H
7H
Homozygote ISR 42-8
Heterozygote
Homozygote Scarlett
Missing data
Figure S1: Map of 47 S42ILs, the map contains 636 BOPA1 SNPs (modified after Schmalenbach et al. 2011).
